# Re-do Boari flap for recurrent ureteric stricture

**DOI:** 10.1590/S1677-5538.IBJU.2020.0491

**Published:** 2020-12-20

**Authors:** Ben V. Sionov, Tarek Taha, Dmitry Preter, Ramzi Salbaq, Dov Engelstein, Alexander Tsivian

**Affiliations:** 1 Tel Aviv University Holon and Sackler Faculty of Medicine E. Wolfson Medical Center Tel Aviv Israel Department of Urologic Surgery, E. Wolfson Medical Center, Holon and Sackler Faculty of Medicine, Tel Aviv University, Tel Aviv, Israel; 2 Bar-Ilan University The Azrieli Faculty of Medicine Galilee Medical Center Israel Department of Urology, Galilee Medical Center, Nahariya, The Azrieli Faculty of Medicine, Bar-Ilan University, Israel

## INTRODUCTION

Repair of ureteral injury may often require excision of long segments and various reconstructive techniques in order to reestablish urinary continuity. Relative long gaps in the distal and mid ureter can be bridged safely, using a bladder psoas hitch (1), and/or a Boari flap (2).

The application of Boari flap in extensive mid-lower ureteral damage has a high success rate of 81-88% (3). Factors such as prior irradiation, de-vascularization, and anastomotic tension contribute to recurrent stricture occurrence. Scares literature deals with the management of Boari flap complications, particularly, ureteral stricture formation at the anastomotic site between the ureter and the Boari flap focusing on the management of the stricture other than the use of bowel segment for reconstruction or autotransplantation (4).

Herein, we present two cases, where a re-do Boari flap reconstruction was performed to manage recurrent benign anastomotic strictures of a re-implanted ureter. To our knowledge, this is the first description of such technique / mode of repair.

## CASE ONE

A 68-year-old male with a solitary kidney, had a history of multiple abdominal surgeries due to adenocarcinoma of sigmoid colon, including left hemi-colectomy and right nephrectomy with adjuvant chemo-radiation therapy. On follow-up, a recurrent tumor was diagnosed at the bowel anastomotic site, including a mass invasion into the distal left ureter. Following the resection of the tumor and the involved segment of the left ureter, a temporary colostomy was created, and ureteroneocystotomy using combined Boari flap and Psoas hitch techniques were applied to reestablish urinary continuity.

**Figure 1:**
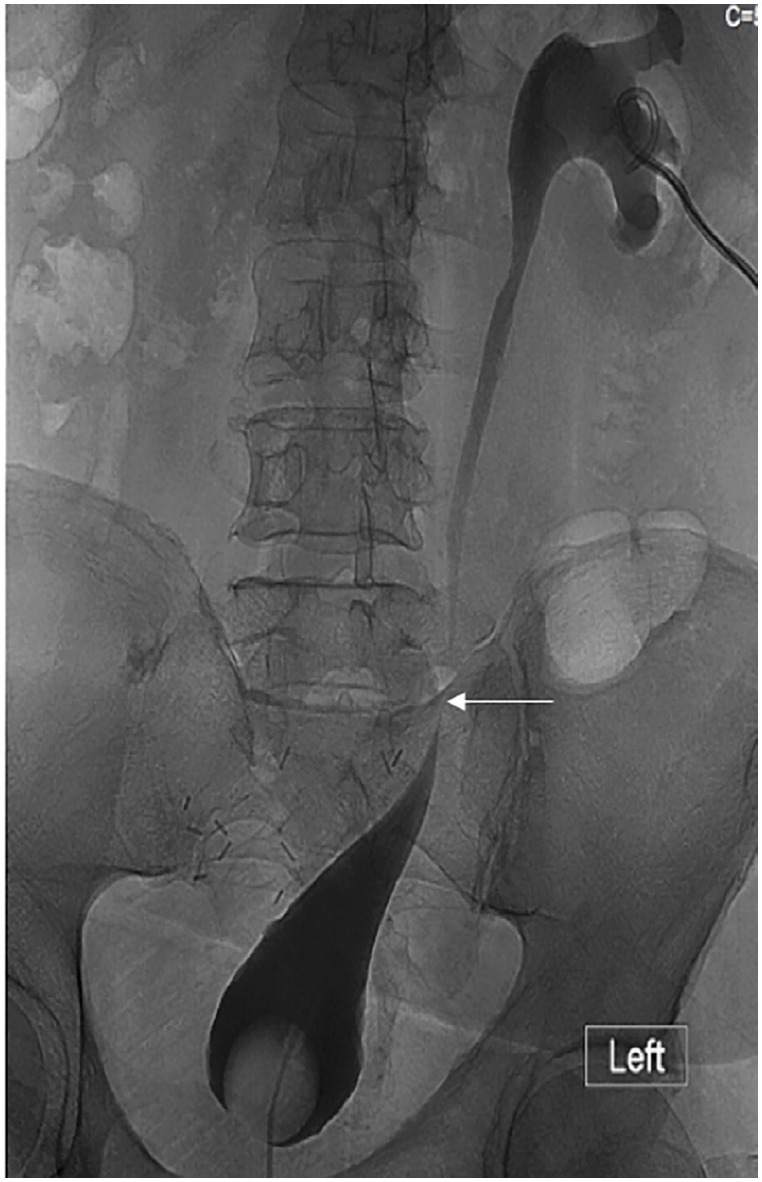
A "rendez-vous" antegrade pyelogram and cystogram via the nephrostomy and urethral catheter demonstrating the ureteral stricture above the primary Boari Flap.

Three years later, laboratory examinations revealed a serum creatinine of 3.4mg/dL (baseline 1.4mg/dL). Physical examination was unremarkable and renal ultrasound (US) demonstrated severe left hydronephrosis. Further antegrade imaging through a percutaneous nephrostomy which was inserted to by-pass the obstruction, confirmed the presence of a 2cm long ureteral stricture above the previous flap formation (Figure-1). Serum creatinine decrease to 1.6mg/dL following insertion of percutaneous nephrostomy. Absence of tumor recurrence was confirmed with PET-CT and cystoscopy.

**Figure 2:**
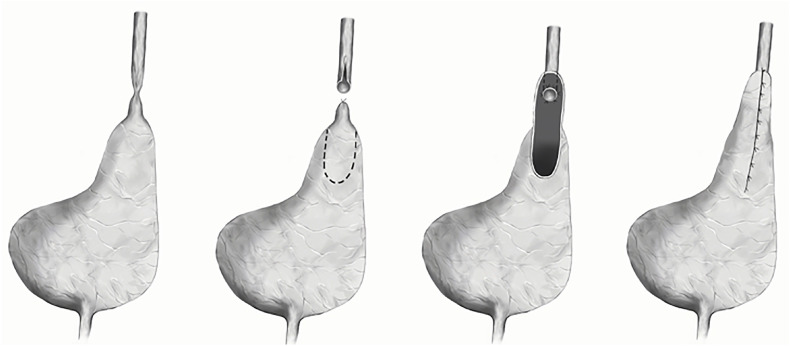
Steps in re-do Boari flap.

**Figure 3:**
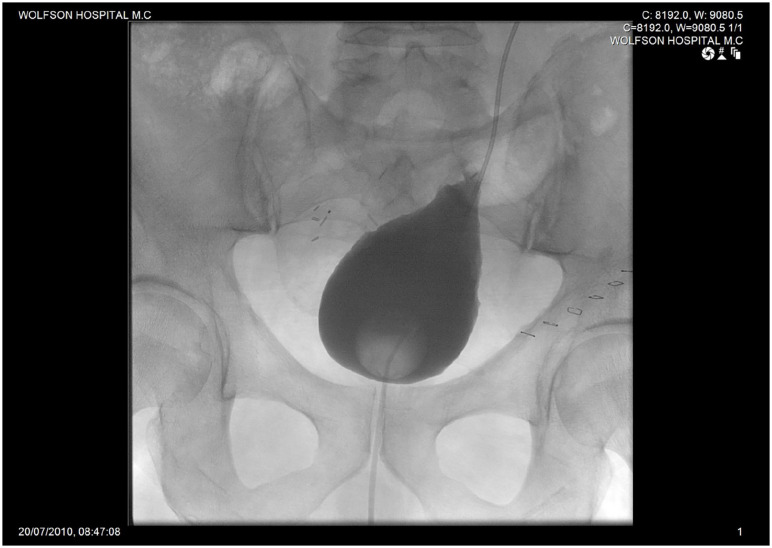
Cystography two weeks following re-do Boari Bladder flap.

A re-do Boari flap was performed, using an open approach: the healthy ureter was transected above the stenotic segment at the level of the previous flap. The ureter was re-implanted to a newly formed Boari flap, which was harvested again from the bladder wall (Figure-2). The postoperative period was uneventful, the urethral catheter and the double J stents were removed 2 and 6 weeks, respectively. Postoperative follow-up imaging showed no urine leak after the surgery before the removal of the urethral catheter (Figure-3). A 10-year follow-up showed no hydronephrosis and no aggravation of the kidney function.

## CASE TWO

A 56-year-old female, with a history of rectal adenocarcinoma, treated with curative low anterior resection and adjuvant radiotherapy. Five year later bilateral mid-distal ureteral stricture was managed with single Boari flap: the two ureteral ends were re-implanted to a single, common channel bladder flap in a Boari fashion.

**Figure 4:**
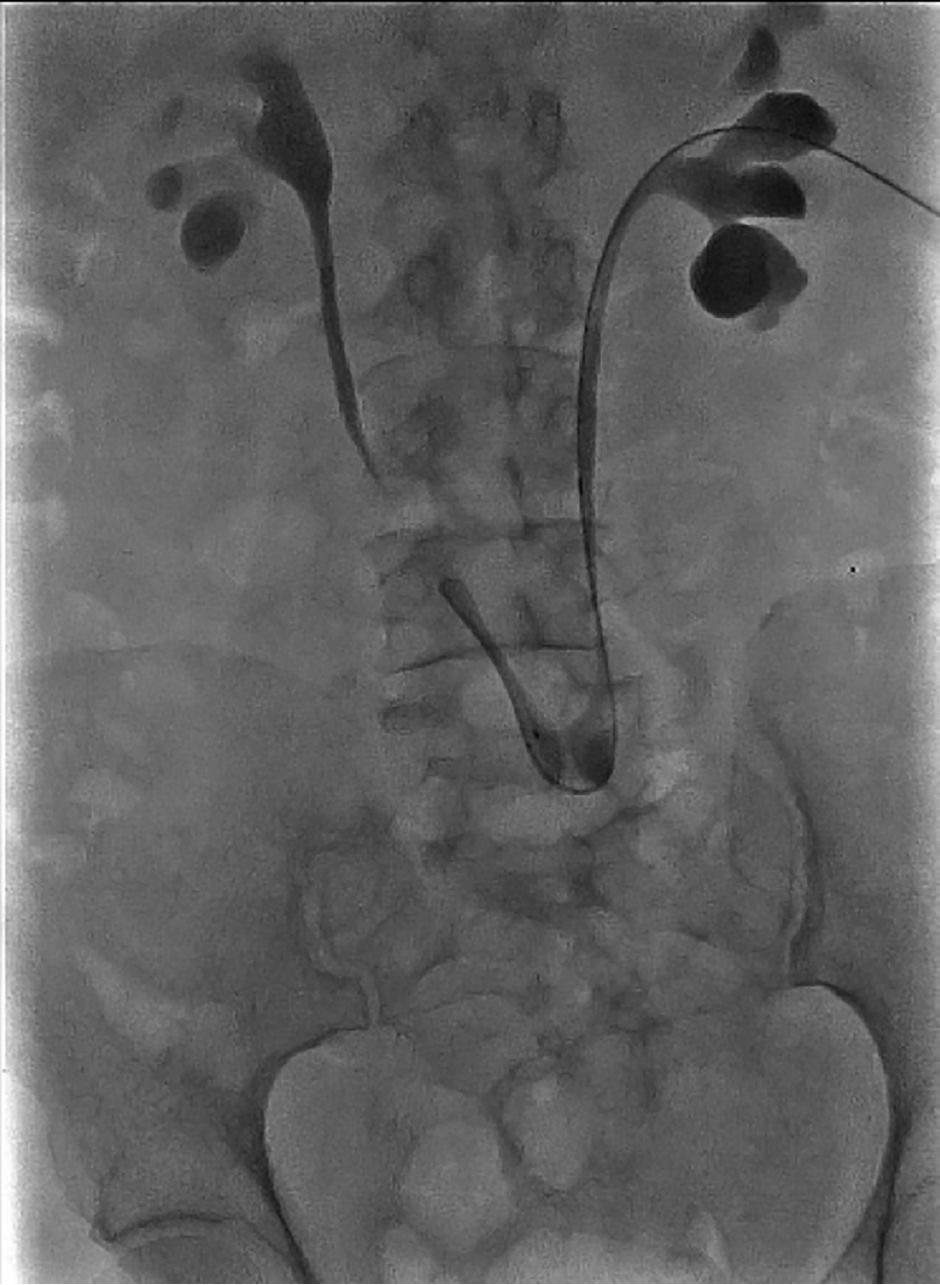
Antegrade pyelogram demonstrating bilateral hydronephrosis without passage of contrast to the bladder.

An year after the surgery, the patient presented with left flank pain, malaise, nausea and vomiting. Bloodwork revealed an acute on chronic renal failure with elevated serum creatinine (6mg/dL). Sonogram showed a bilateral hydronephrosis, larger on the left side, hence a left percutaneous nephrostomy was performed. Further radiological evaluation revealed that the bilateral hydronephrosis was due to a benign anastomotic stricture of the Boari flap, not amenable to an attempt to pass a guidewire (Figure-4).

**Figure 5:**
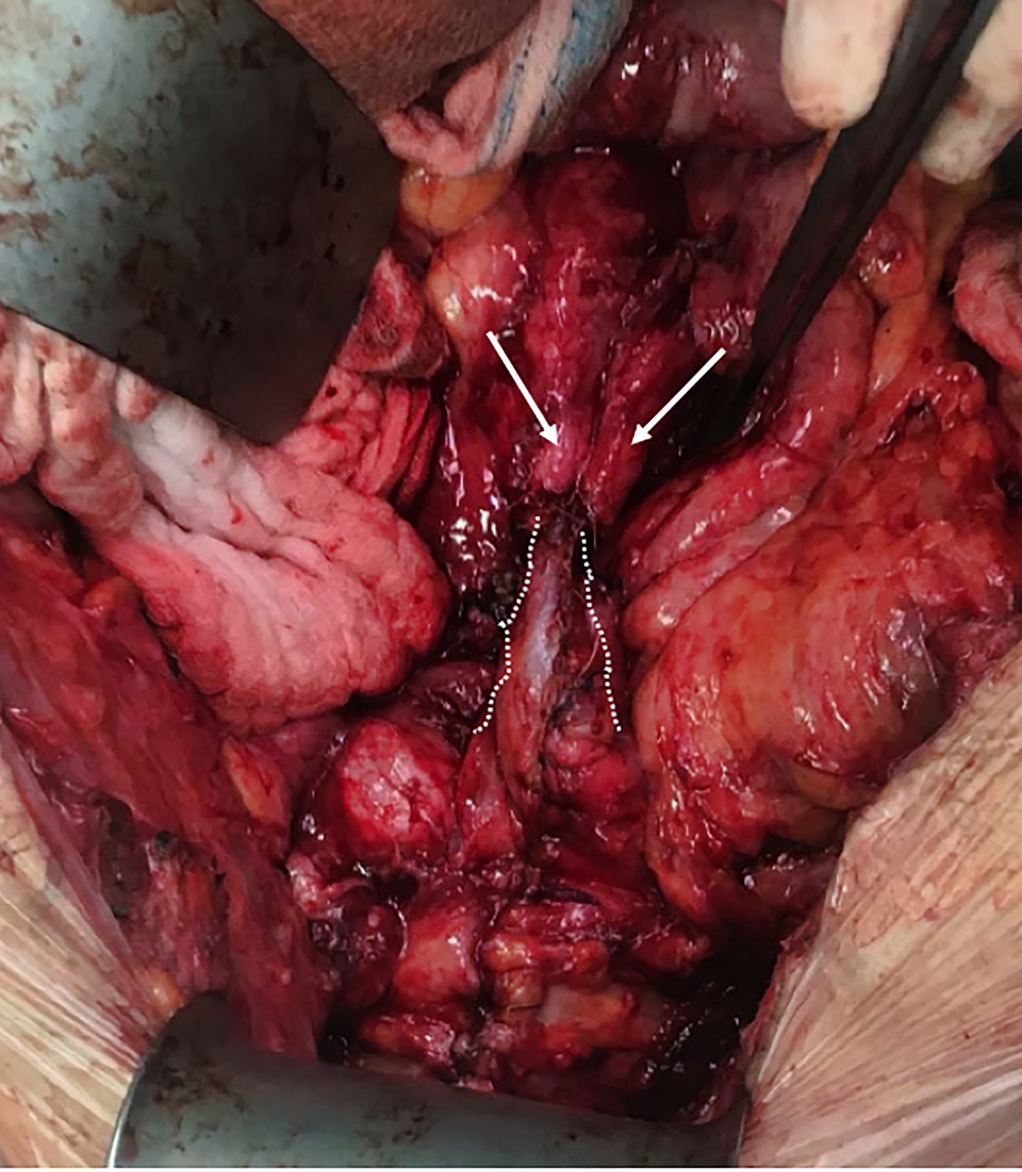
Ureters (arrows) reimplanted to a common channel Boari flap (dotted line).

Here again, from the various available reconstructive solutions techniques, a re-do Boari flap was elected. Intraoperatively, in the anatomically distorted pelvis, the anastomotic stricture was dissected, exposed and resected, a new Boari flap from the bladder wall was reconstructed to where both healthy distal ends of the ureters were re-implanted (Figure-5). Postoperative period was uneventful, urethral catheter was removed following cystography 2 weeks after the surgery. At two years post-operatively, a mild hydronephrosis but a patent Boari flap anastomosis was noticed, with a steady serum creatinine values around 2.8mg/dL and no further renal deterioration.

## DISCUSSION

The options available to treat a large ureteric defect are transureteroureterostomy, psoas hitch, Boari flap, ileal segment replacement for the lost ureter and renal autotransplantation. Boari flap is a reliable technique to reconstruct ureteral defect regardless of their site (5). The number of reported patients treated with a Boari flap is not significant, yet the results are excellent if a well-vascularized, tension-free flap is used (6).

Reconstruction of the ureter using the Boari technique may be associated with both early and late complications. Early complications include urinary leakage and bleeding. It has been suggested that the placement of a double-J stent may prevent postoperative urinary leakage and promotes healing. Commonly described late complications are persistent reflux and stricture of the anastomosis.

Pearson et al.. (7) reported on one patient who developed ureteric obstruction following Boari flap without outlining his management and outcome.

In a complexed ureteral reconstruction, the use of gastrointestinal segments has been described with good functional results, when available urinary tract cannot be used (8,9). The use of ileum for ureteral substitution in our specific cases was rejected due to the patient's history of gastrointestinal malignancy, bowel resections, previous radiotherapy, and the intent to avoid negative metabolic derangements (10).

Despite technical difficulties in dissecting and exposing the previously re-implanted ureter(s) and taking into consideration the patient's relatively normal bladder volume, we decided to form a new Boari flap. The re-do flap was harvest from the existing flap in one case (Figure-2), and a new one in the second case without compromising the bladder capacity. Perioperative care of the first and second approaches were similar in relation to the time of urethral catheter and double J removal. This technique was carried out successfully, without added morbidities and with excellent anatomical (Figure-3) and functional outcome, on follow-up at 10 in the first case and 2 years in the second patient.

There is a dearth literature on the management of recurrent anatomic stricture after Boari flap repair. To our knowledge, these two reports are the first to describe re-do Boari flap. This technique is suggested as a different facilitated method for management of recurrent Boari flap anastomotic stricture, without the use of a bowel segment or augmentation, hence avoiding additional morbidity.

## CONCLUSION

Repeated Boari flap is a reliable reconstructive solution for anastomotic ureteral stricture in selected patients and may be added to the urologist's surgical armamentarium.
